# Measuring the relationship between body mass index and depression among Saudi adult population: A nationwide cross-sectional study

**DOI:** 10.1371/journal.pone.0293799

**Published:** 2023-11-16

**Authors:** Mohamed O. Nour, Tamara Abdulrahman Hafiz, Khulud K. Alharbi

**Affiliations:** 1 Department of Health Promotion and Education, Faculty of Public Health & Health Informatics, Umm Al-Qura University, Makkah, Kingdom of Saudi Arabia; 2 Department of Public Health and Community Medicine, Damietta Faculty of Medicine, Al-Azhar University, Cairo, Egypt; 3 Department of Health Services Management, Faculty of Public Health & Health Informatics, Umm Al-Qura University, Makkah, Kingdom of Saudi Arabia; Tehran University of Medical Sciences, ISLAMIC REPUBLIC OF IRAN

## Abstract

**Background:**

The prevalence of obesity and depression shows an accelerating trend with increased risk of morbidity and disability. The exact underlying relationship between them is unclear. We aimed to evaluate the prevalence of body mass index (BMI) and depression and their associations in a large sample of Saudi adults.

**Methods:**

We administered a nationwide cross-sectional web-based survey using a snowball sampling method among Saudi adults aged 18–60 years. We used a validated Arabic version of Beck’s Depression Inventory-II (BDI-II) for depression assessment. We classified BMI into underweight, normal weight, overweight, and obesity. We used logistic regression analysis to determine the factors associated with depression.

**Results:**

Among 4,683 Saudi adults, different grades of depression were present in 43.3%, most (25.2%) with a mild condition. Overweight and obesity were present in 26.4% and 21%, respectively. We found a positive association between BMI and BDI-II score (ρ = 0.14, *p* = 0.006). BMI was significantly higher among those who were older, males, married, living in the Eastern region of Saudi Arabia, educated at a pre-university level, employed, at high family-income levels, smokers, and people with chronic diseases. Depression score was significantly higher among married, non-employees, non-smokers, people with chronic diseases, and those with BMI ≥ 25 kg/m^2^. Non-smoking, presence of chronic diseases, and being overweight or obese were significantly associated with depression.

**Conclusions:**

Saudi adults were suffering from different grades of depression, overweight, and obesity. A positive association between BMI and BDI-II score was observed. Depression score did not differ by age, sex, geographical region, educational level, or family income. Non-smoking, presence of chronic diseases, and being overweight or obese were significantly associated with depression. Further longitudinal research is required to understand the factors underpinning causal relationships between BMI and depression, the subgroups’ variation, and mediating strategies.

## Introduction

The prevalence of both obesity and depression shows an accelerating trend across all ethnic groups and among adults of both sexes [1]. Overweight and obesity, according to the World Health Organization (WHO), are defined as “abnormal or excessive fat accumulation that may impair health.” Body mass index (BMI) is a simple weight-for-height index that is used to classify obesity and overweight in adults. However, it should be used only as a general guide because it may not reflect the same level of obesity in various people [2].

Depression is described as “a mental condition characterized by chronic melancholy, lack of interest in, desire for, and enjoyment of formerly enjoyable activities, as well as possible sleep and eating issues” [3]. Inability to care for oneself, incapacity, and suicide are primarily brought on by depression. Furthermore, both obesity and depression are associated with short- and long-term consequences for population health and economy including increased risk of disability, chronic diseases, mortality rates, and health-care expenses, and a decline in quality of life, life expectancy, and productivity [4, 5].

WHO has estimated that there are about 1.3 billion overweight adults, over 600 million obese persons, and 350 million people from all age groups who suffer from depression [6, 7]. So far, research has revealed an association between obesity and depression, as the prevalence of depression among obese people is more than twice the prevalence of depression in persons of normal weight [8, 9]. Some researchers have argued that obesity leads to depression [10, 11], and others have claimed that depression leads to weight gain and obesity [12]. Taken together, the research suggests a bidirectional relationship, and sociodemographic characteristics may modulate the association between BMI and depression [13].

The precise underlying relationship between obesity and depression is uncertain. Three hypotheses have been proposed: positive association (higher depression is correlated with more obesity) [14, 15], negative association (higher depression is correlated with lower obesity) [16, 17], and an absence of association [18, 19]. In addition, a nonlinear (U-curved) association—meaning that both obesity and underweight are associated with depression—can be expected [20, 21]. To our knowledge, there are no research studies at the national level in Saudi Arabia that measured the relationship between BMI and depression or that investigated whether the trend was linear or nonlinear. Therefore, we aimed to measure the prevalence of obesity and depression at a national level, and to explore their associations in a large sample of Saudi adults.

## Materials and methods

### Study design

This study was conducted using a cross-sectional web-based survey design.

### Sampling and sample size

The study used a snowball sampling method and calculated the sample size with the Raosoft sample size calculator (http://www.raosoft.com/samplesize.html). The minimum required sample size was 1,037 based on the following factors: a 99% confidence interval, a standard deviation set at 1.96, a 4% margin of error, an expected response of 50%, and a total Saudi-adult population of 13,110,901 (aged 18–60 years, representing about 70% of the total population) (according to issue number 55 of the Statistical Yearbook published by General Authority for Statistics, 2019 (https://www.stats.gov.sa/ar/1007-0) and the updated mid-2020 report (https://www.stats.gov.sa/en/43)]. To avoid bias, all eligible respondents with complete responses during the survey period from January through April 2022 were enrolled.

### Population and inclusion criteria

The study included members of the general adult Saudi population, aged 18–60 years, of both sexes, residing in all administrative districts within the Kingdom (Western, Northern, Southern, Central, and Eastern regions), not suffering from chronic mental or psychological illness, and not having BMI below 14 or above 60 (considered outliers). In a trial to achieve a proportional nationwide representative sample, we considered weighing based on different age groups, gender, and population distribution within different regions. This approach aimed to align with data obtained from the General Authority for Statistics (with accepted ±5% variation for each subgroup). In total, 5,896 participants took part in the survey, of which 4,683 (79.4%) matched the criteria for inclusion.

### Preparing the study instrument

We used an Arabic version of the validated Beck’s Depression Inventory-II (BDI-II) for depression assessment. The Arabic version has been previously validated for use in Arab populations [22–24]. The BDI-II is self-administered and takes about 5–10 minutes; the recall period is 2 weeks. It includes twenty-one items on a 4-point scale ranging from zero (no symptoms) to 3 (severe symptoms). Scoring is calculated by adding the highest ratings for all twenty-one items. The minimum and maximum scores are zero and 63, respectively. Higher scores reflect greater symptom severity. In non-clinical subjects, scores of zero–13 indicate no depression and 14–19, mild depression. Scores above 20 indicate depression: 20–28 (moderate depression) and 29–63 (severe depression). The validity and reliability of BDI-II across different populations and countries have been examined in many studies, revealing good internal consistency (Cronbach’s alpha 0.91) and high test-retest reliability (Pearson *r* = 0.93) [25, 26].

We measured BMI by dividing weight in kilograms (kg) by height in meters squared (m^2^). Weight and height were both self-reported. According to WHO criteria, we divided weight into four categories: underweight (BMI < 18.5 kg/m^2^), normal weight (BMI 18.5–24.9 kg/m^2^), overweight (BMI 25.0–29.9 kg/m^2^), and obesity (BMI > 30.0 kg/m^2^) [27].

The questionnaire was self-reported and included the following:

■ Sociodemographic characteristics such as age, gender, geographical district, nationality, social status, occupation, education, and income.
■ Smoking habit and presence of chronic illness such as high blood pressure, diabetes, cardiovascular disease, asthma, chronic kidney disease, chronic liver disease, high cholesterol, cancer, and others.
■ Presence of chronic mental/psychological illness (they were 22 subjects, accounted for less than 0.5%, and excluded from analysis).
■ Weight in kilograms and height in centimeters (to calculate the BMI, and those with BMI below 14 (two subjects) or above 60 (three subjects) were excluded from analysis).
■ The twenty-one items of BDI-II.

We distributed the questionnaire via various means of communication on the internet and social media platforms such as Twitter, WhatsApp, and Telegram. Participants were encouraged to share the survey link using a snowball sampling technique. In addition, personal communications contributed to rapid distribution of the survey. The decision to participate was completely voluntary, and respondents were not provided with any form of incentives for participation or sharing links to the survey. By clicking the relevant link, every participant had the opportunity to access the questionnaire and answer the questions.

### Ethical considerations

Ethical approval was obtained from the Bioethics Committee at Umm Al-Qura University (HAPO-02-K-012-2022-03-1000). Prior to commencing the online survey, participants were provided with an initial cover page containing an information letter. This letter emphasized the study’s objectives and the basic items involved, ensuring clarity for the participants. Written informed consent was obtained electronically from all participants on the cover page of the questionnaire, indicating their voluntary participation and the option to withdraw from the study at any time without providing a reason. So, each participant would only be able to start the survey after clicking on the “Accept to participate” icon on the cover page. It is important to note that this study strictly adhered to the inclusion of adult participants only (aged 18–60 years), and no minors were involved.

### Statistical analysis

We used the software program SPSS (IBM SPSS, V 25.0, Armonk, NY: IBM Corp., USA). For descriptive statistics: we used the mean ± SD and median (interquartile range [IQR]) for quantitative variables and frequency and percentage for qualitative variables. We examined numerical data for normality using the Shapiro–Wilk test. We used the Mann-Whitney or Kruskal Wallis tests for continuous non-normal distribution variables (BMI and depression score). We used the Spearman correlation coefficient to correlate both BMI and depression score. The specific association between BMI and depression was further explored. Both crude and adjusted Odds ratios (ORs) with 95% confidence intervals (95% CIs) were considered using three models; Model 1 (referred to as unadjusted) including the total sample, Model 2 (adjusted for age, gender, and socio–demographics (marital status, educational level, occupation, family income), and Model 3 (adjusted for all covariates to rule out the confounding effects of population heterogeneity; age, gender, socio–demographics (marital status, educational level, occupation, family income), lifestyle factors (smoking habit), and presence of chronic diseases, referred to as “adjusted for all factors”). Factors associated with depression (dependent variable) (score ≥ 14) were tested by logistic regression analysis where OR and 95% CI were calculated for each independent variable. A significant level of *p* < 0.05 (two-tailed) was considered.

## Results

The study included 4,683 adult Saudi participants filling the inclusion criteria with mean age 29.9 ± 11.1 years, ranging from 18–60 years, and the majority (62.7%) were from the 18–30 age group. More than half of participants (56.8%) were males, 40.5% were married, and 42.4%, 22.2%, 20.5%, 7.9% and 7% were living in the Western, Southern, Central, Northern, and Eastern regions of Saudi Arabia, respectively. Only 3.2% had primary or middle school education, while the majority (70%) were university graduates; 6% had postgraduate studies, 42.4% were students, 24.1% were working for the government, and 29.9% had family income less than 5,000 riyals per month. A total of 643 (13.7%) were smokers, and 727 (15.5%) had chronic diseases. The most common chronic conditions, either individually or in combination with others, included asthma, 266 (5.7%), hypertension, 240 (5.1%), and diabetes, 221 (4.7%) (Table 1).

**Table 1:** General characteristics of the studied sample.

Variables		N = 4683	%
**Age (years)**	18–30 years	2,936	62.7
	31–40 years	845	18.0
	41–50 years	622	13.3
	51–60 years	280	6.0
**Gender**	Male	2,659	56.8
	Female	2,024	43.2
**Marital status**	Married	1,896	40.5
	Unmarried ^**a**^	2,787	59.5
**Region**	Western	1,984	42.4
	Central	961	20.5
	Eastern	328	7.0
	Southern	1,039	22.2
	Northern	371	7.9
**Educational level**	Primary/Middle school	148	3.2
	High school /Diploma	980	20.9
	University	3,276	70.0
	Postgraduate	279	6.0
**Occupation**	Governmental sector	1,127	24.1
	Private sector	439	9.4
	Retired	177	3.8
	Student	1,984	42.4
	Do not work	956	20.4
**Family income per month (riyal)**	Less than 5,000 riyals	1,401	29.9
	5,000–10,000 riyals	1,372	29.3
	More than 10,000 riyals	1,910	40.8
**Smoking habit**	Smoker	643	13.7
	Ex-smoker ^**b**^	171	3.7
**Chronic diseases**	Yes ^**c**^	727	15.5

^**a**^: Includes single (2,587; 55.2%), divorced (153; 3.3%), and widow (47; 1%).

^**b**^: Includes those who were smokers then stopped for several months or more.

^**c**^: Includes 543 (11.6%) had one chronic disease, 143 (3.1%) had two chronic diseases, 41 (0.9%) had three or more chronic diseases.

**Fig 1:**
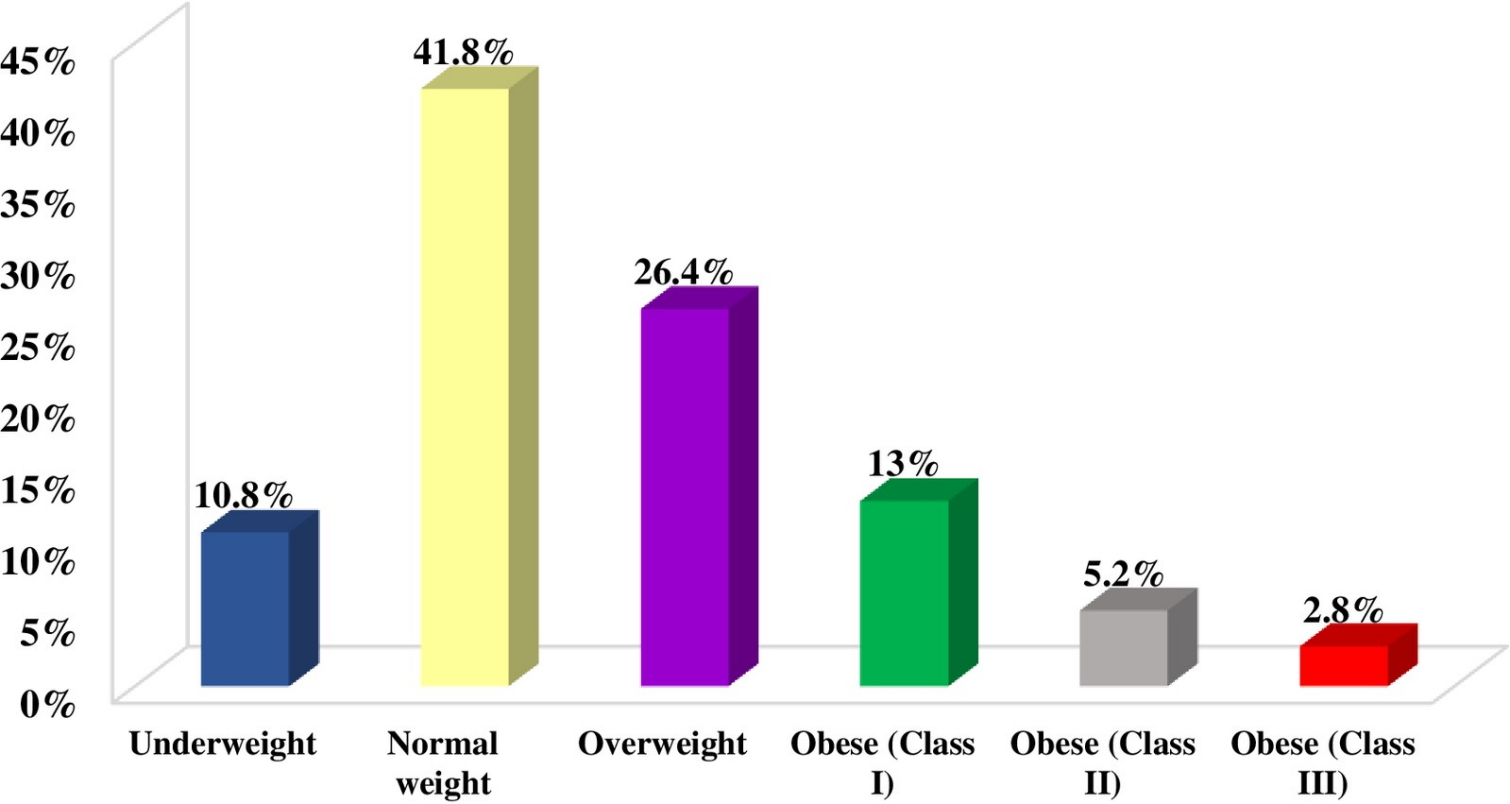
Classification of Saudi adults according to BMI.

**Fig 2:**
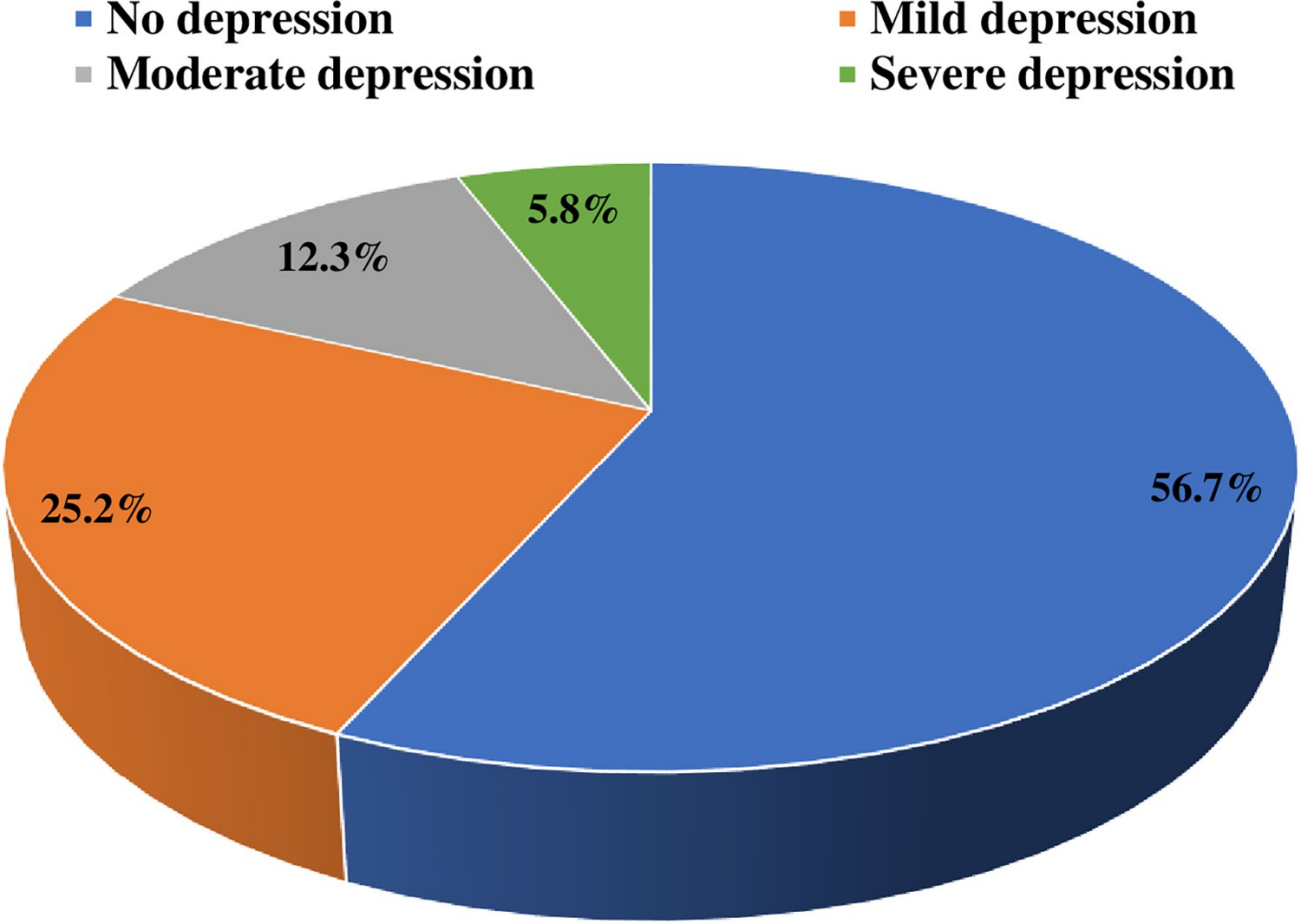
Depression among Saudi adults according to the Beck’s Depression Inventory-II (BDI-II).

**Fig 3:**
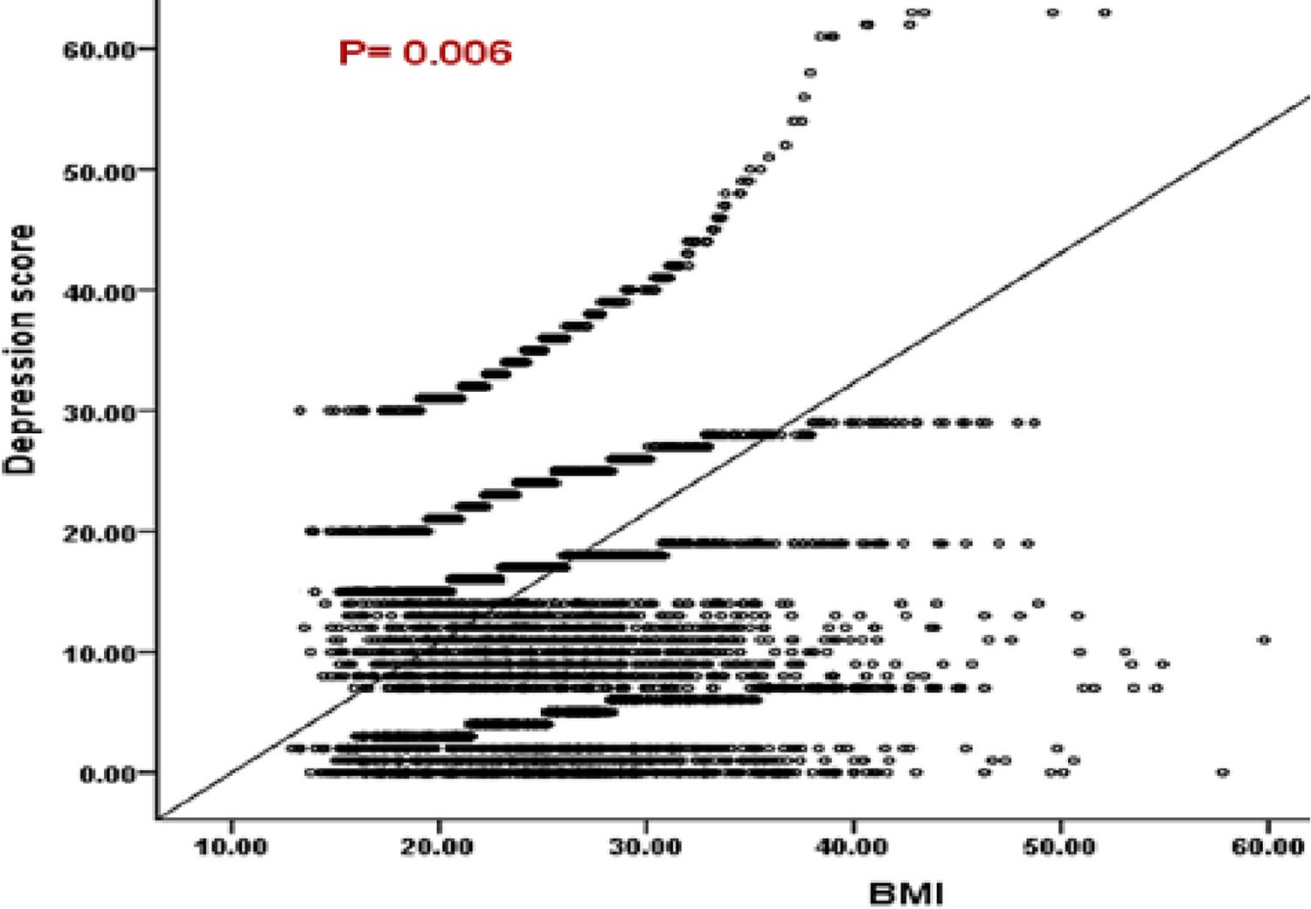
Correlation between BMI and depression score among Saudi adults.

The mean BMI of participants was 25.6 ± 7.4, ranging from 14–60 kg/m^2^, and the median (IQR) was 24.6 (21.1–29) kg/m^2^. A total of 505 (10.8%) participants were underweight, 1,957 (41.8%) were normal weight, and 1,237 (26.4%) were overweight, while 984 (21%) suffered from different grades of obesity (Fig 1). The mean depression score of participants according to BDI-II was 11.3 ± 10.3 and the median (IQR) was 9 (3–17). A total of 2,029 (43.3%) were suffering from different grades of depression ranging from 1,179 (25.2%) with mild depression, 576 (12.3%) with moderate depression, and 274 (5.8%) with severe depression (Fig 2). We found a significant positive correlation between BMI and depression score (Spearman’s rho = 0.14, *p* = 0.006) (Fig 3).

Both BMI and depression score were tested against different study variables (that were dichotomized when appropriate). BMI was significantly higher among participants who were older, male, married, living in the Eastern region, working, and smokers, in addition to those with higher family income, pre-university education, and chronic diseases. Depression score was significantly higher among those who had chronic diseases and were married, non-workers, non-smokers, and those suffering from overweight/obesity with BMI ≥ 25 kg/m2. Depression score did not differ by age, sex, geographical region, educational level, or family income (Table 2).

**Table 2:** Relation between BMI and depression score with different characteristics of participants.

Variables		BMI		Depression score	
		Median (IQR)	*P*-value	Median (IQR)	*P*-value
**Age (years)**	18–40 years	23.5 (20.4–27.5)	<0.001*	9 (3–17)	0.066
	41–60 years	29 (26–32.7)		8 (3–17)	
**Gender**	Male	25.7 (22.2–29.6)	<0.001*	8 (3–17)	0.339
	Female	24.2 (20.6–28.7)		9 (3–17)	
**Marital status**	Married	27.3 (24–31.3)	<0.001*	9 (3–17)	0.015*
	Unmarried	22.7 (19.6–26.8)		8 (3–17)	
**Region**	Western	24.4 (20.6–28.8)	<0.001*	8 (3–17)	0.053
	Central	25.2 (21.6–29.9)		9 (3–17)	
	Eastern	25.9 (21.6–30)		9 (3–18)	
	Southern	24.3 (20.9–28.4)		9 (3–18)	
	Northern	21.9 (26–28.4)		10 (3–18)	
**Education**	Pre-university	25.6 (21.5–30.4)	<0.001*	9 (3–18)	0.271
	University/Postgraduate	24.4 (20.9–28.6)		9 (3–17)	
**Occupation**	Working	26.9 (23.4–31.1)	<0.001*	8 (3–16)	0.048*
	Do not work	23.5 (20.1–27.7)		9 (3–17)	
**Family income/Month**	< 5000 riyals	23.7 (20.2–27.6)	<0.001*	9 (3–17)	0.518
	≥ 5000 riyals	25.2 (21.6–29.4)		9 (3–17)	
**Smoking habit**	Smoker	25.8 (21.8–29.9)	<0.001*	6 (2–13)	< 0.001*
	Non-smoker	24.5 (20.9–28.8)		9 (3–18)	
**Chronic diseases**	Yes	27.4 (23.1–32)	<0.001*	11 (4–21)	< 0.001*
	No	24.2 (20.8–28.4)		8 (3–17)	
BMI (kg/m^2^)	BMI < 25			8 (3–16)	< 0.001*
	BMI > 25			9 (5–18)	

*: Significant.

The association between BMI and depression was explored. First, BMI was entered into the model as a continuous variable and then as a categorical variable (considering subjects with normal weight as reference group) using both unadjusted and adjusted models. The ORs showed significant association between BMI and depression in the model adjusted for all important confounders with each 5 unit increase in BMI was associated with an OR of 1.32 (1.25–1.43, P = 0.003). Further, the specific association between BMI categories and depression showed that the ORs for depression in underweight, overweight, and obesity groups were 1.39 (1.19–1.63), 1.73 (1.49–2.00), and 1.91 (1.56–2.34) respectively. After adjustment of all factors in Model 3, the ORs for depression in underweight, overweight, and obesity groups were 1.49 (1.26–1.76), 1.76 (1.51–2.05), and 1.92 (1.56–3.36) respectively with higher ORs for depression among obese then overweight participants and the trend was significant in all (*P* < 0.05). (Table 3)

**Table 3:** Association between BMI categories and depression.

Variables	No. of cases	Model 1 OR (95% CI)	Model 2 AOR (95% CI)	Model 3 AOR (95% CI)
**BMI (per 5-unit increase)**	4683	1.61 (1.36–2.08)	1.41 (1.18–1.70)	1.32 (1.25–1.43)
*P* for trend		<0.001*	0.005*	0.003*
**BMI (categories)**				
Normal weight	1957	Reference (1.00)	Reference (1.00)	Reference (1.00)
Underweight	505	1.39 (1.19–1.63)	1.52 (1.20–1.97)	1.49 (1.26–1.76)
Overweight	1237	1.73 (1.49–2.00)	1.68 (1.35–2.34)	1.76 (1.51–2.05)
Obesity	984	1.91 (1.56–2.34)	1.75 (1.41–2.47)	1.92 (1.56–3.36)
*P* for trend		<0.001*	0.001*	<0.001*

BMI: body mass index; OR: Odds ratio; AOR: Adjusted odds ratio.

Model 1: Unadjusted.

Model 2: Adjusted for age, gender, marital status, educational level, occupation, and family income.

Model 3: Adjusted for all factors (Model 2 plus smoking habit and presence of chronic diseases).

* Significant.

We further dichotomized the depression score into no depression (score zero–13) and depression (score ≥ 14). We analyzed variables in univariate analysis that significantly affect depression score with a multinomial logistic regression to explore independent variables associated with depression. Non-smoking (OR = 1.76; CI: 1.46–2.11), presence of chronic diseases (OR = 1.6; CI: 1.34–1.88), and BMI ≥ 25 kg/m^2^ (overweight/obesity) (OR = 1.41; CI: 1.24–1.6) were significantly associated with depression among Saudi adults (Table 4).

**Table 4:** Multinomial logistic regression of factors associated with depression among Saudi adults.

Independent variables	Coefficient	OR	CI (95%)	*P*-value
**Marital status (married)**	0.02	0.98	(0.86–1.12)	0.810
**Occupation (do not work)**	0.05	0.95	(0.83–1.09)	0.482
**Smoking habit (non-smoker)**	0.56	1.76	(1.46–2.11)	< 0.001*
**Chronic diseases (yes)**	0.47	1.6	(1.34–1.88)	< 0.001*
BMI (kg/m^2^) (BMI ≥ 25)	0.34	1.41	(1.24–1.6)	< 0.001*

OR: Odds ratio, CI: Confidence interval.

*: Significant.

## Discussion

The study aimed to determine the prevalence of BMI categories and depression, and to explore their associations in a large sample of Saudi adults. We found that 43.3% of Saudi adults were suffering from different grades of depression ranging from mild (25.2%), moderate (12.3%), and severe (5.8%). These results are comparable to those reported by Al-Qadhi et al., in which 49.9% of Saudi adults who routinely visited primary care centers exhibited depressive symptoms. of which 31%, 13.4%, 4.4%, and 1% were mild, moderate, moderate-severe, and severe, respectively [28]. However, the prevalence of depression in our study was higher than that reported among the general population in the Jazan region in Saudi Arabia during the COVID-19 pandemic, with a rate of nearly 26% [29] and higher than that reported by Alamri et al., in which 28.9% of the general Saudi population reported experiencing any depressive symptoms, of which 11.8% were mild, 10.1% were moderate, and 7% were severe [30].

Our findings related to the prevalence of depression were higher than those reported by Alzahrani et al., who investigated the prevalence of common mental disorders, including depression related to the COVID-19 pandemic, among the Saudi general population by a systematic review and meta-analysis. They found that the overall prevalence of depression was 30% (95% CI: 22% to 38%, I2 = 99.58%) [31]. The variation in prevalence could be attributed to different population characteristics, the variable consequences of the COVID-19 pandemic, and the use of different depression screening scales in the studies.

Slightly less than half of our participants (47.4%) suffered from being overweight and obese (26.4% and 21%, respectively), while about 10.8% were underweight. The overall national-level prevalence of overweight/obesity among Saudi adults in the last two decades showed a decreasing trend, with 36.9% overweight and 35.6% obesity between 1995 and 2000 [32], 28.7% obesity in 2013 [33], and 24.7% obesity in 2020 [34]. However, the international figure reported by the 2019 World Health Survey found the overall prevalence of overweight/obesity among Saudi adults to be 58.4% [35]. The patterns of Saudi food consumption and physical activity are among factors contributing to prevalence variations [36].

Both overweight/obesity and depression can be costly to the individual and the health-care system. Thus, there is a need to understand the causal relationships between these two disorders to enhance implementation of preventive measures. To the extent that some individuals have depression and obesity, obesity prevention efforts could help reduce the prevalence of depression, and programs targeting depression could have the added benefit of reducing the prevalence of obesity [37].

Our findings add to the growing literature suggesting that obesity/overweight is associated with depression among adults, and they revealed a significant positive association between depression and BMI (more severe depression is associated with increased BMI), which is consistent with other studies [14, 15, 38, 39] but inconsistent with other studies that reported negative association [16, 17], no association [18, 19], or a nonlinear (U-curved) association [20, 21].

Although most of the studies reported a relationship between both depression and obesity, understanding the meaning of this relationship is complex and unclear, and a significant relationship cannot necessarily reflect causality. Several factors can affect this relationship, such as sex, ethnicity, genetics, biology, culture, unhealthy lifestyle, poor self-esteem, the stigma attached to obesity, emotional eating, the presence of associated chronic conditions, and the use of psychiatric medication [13, 38–43]. The occurrence of obesity as a consequence of chronic illnesses is another concern that may affect the relationship. Improving patients’ quality of life, lifestyle, emotional management, and psychological support can be useful in both obesity and depression intervention.

Almarhoon et al. recently investigated the relation between depression and obesity among adults in the Eastern region of Saudi Arabia. They found that the association was most prominent in young adults (18 to 25 years), in being single, in the presence of associated chronic or psychiatric conditions, in having a BMI > 30 for 10 years or more, and in taking regular medications [44].

According to our findings, not smoking, having a chronic disease, and being overweight or obese were significantly associated with depression among Saudi adults. Other studies have shown that the association between depression and BMI is gender dependent [39], and others have confirmed the role of socioeconomic status [45].

In summary, there is a consensus that obesity/overweight is associated with increased depression. However, further longitudinal research is required to better understand the factors that underlie the causal relationships between them, the subgroups among whom they exist, and mediating strategies.

To our knowledge, this is the first nationwide study in Saudi Arabia that has measured the relationship between depression and BMI. The findings of our study underscore the importance of addressing obesity as a significant public health concern. The prevalence of obesity and its associated negative consequences, including increased risk for depression, highlight the need for comprehensive and more targeted health education programs on obesity. When developing such programs, it is essential to consider the cultural diversity within the local community and the influence of negative behaviors and false beliefs that affect the ability of society to maintain physical and psychological well-being. These factors have led to an increase in the prevalence of depression and a delay in taking the necessary measures and timely medical interventions for cases of weight disorders, which may pose a threat to individuals and society.

## Study limitations

Our findings should be viewed with caution as the study has several limitations. (1) Causal inferences and the reciprocal effects are difficult to assess in cross-sectional studies. (2) The snowball non-random method carries a possible sampling bias. (3) There is a potential selection bias when using a web-based survey (being accessible to web users only with possible overrepresentation of health-oriented and more interested individuals). (4) There may be recall bias of self-reported data and social desirability bias especially for weight and height measurements, presence of chronic illness, and being suffering from chronic mental/psychological illness. The study employed self-reporting as a practical and efficient method to identify and exclude individuals with chronic mental or psychological illness from the analysis. It is important to note that the study did not specifically investigate the relationship between depression and individual chronic diseases but instead focused on the overall relationship between depression and chronic diseases as a collective category. These limitations should be taken into consideration when interpreting the findings of the study. (5) Non-Saudi residents within the Kingdom and the older age group were not included. In addition, a related concern here is the overrepresentation of young adults and those with a university degree that might affect the generalizability of findings. (6) BDI-II is valid for measuring depressive symptoms but cannot be used as a diagnostic instrument for clinical depression. (7) Categorization of continuous variables, namely BMI and depression, is a concern that affects our findings with possible bias, loss of power, missing non-linear relationship, and incomplete correction for confounding factors. Also, possible over-adjustment in multi-variable adjusted models may be a concern (8) Depression episodes may occur during anyone’s life span, so lifetime prevalence is needed to study the association. (9) Residual confounding caused by non-measured covariates might have occurred, such as the concurrent impacts of COVID-19 and other psychological, behavior, dietary, and lifestyle factors. (10) Variability of geographical, cultural, and ethnic backgrounds of other populations may affect the findings. Furthermore, cross-cultural differences in the symptomatology and experience of depression should not be neglected.

## Conclusions

A remarkable percentage of Saudi adults were suffering from different grades of depression (43.3%), overweight (26.4%), and obesity (21%). A positive association was found between BMI and depression score. Depression score did not differ by age, sex, geographical region, educational level, or family income. Non-smoking, having chronic diseases, and being overweight/obese were significantly associated with depression. Further longitudinal research is required to better understand the factors underpinning the causal relationships between BMI and depression, the subgroups among whom they exist, and mediating strategies.

## Supporting information

S1 Data (XLSX)
